# Changes in behavior patterns or demographic structure? Re-estimating the impact of higher education on the average age of the first marriage

**DOI:** 10.3389/fpsyg.2023.1085293

**Published:** 2023-01-27

**Authors:** Ting Lai, Yiheng Huang, Jinwu Xiong

**Affiliations:** ^1^China Center for Special Economic Zone Research, Shenzhen University, Shenzhen, China; ^2^Business School, China University of Political Science and Law, Beijing, China

**Keywords:** higher education, the average age of the first marriage, demographic structure, behavior patterns, macro factors

## Abstract

During the last few decades, China implemented college enrollment expansion to accelerate the process of urbanization. However, most existing papers blaming that receiving higher education may delay people choosing to enter the age of first marriage, which in turn results in the age of the population. In this paper, we argued that the previous papers confused the total impact of higher education on the average age of the first marriage with the influence on individual’s behavior change, and thus led to overestimating the delayed effect of higher education on the age choosing behavior of first marriage. The present paper re-estimated the impact of higher education on the average age of the first marriage in China with both extensive and intensive margins using the duration model and qualified the pure effect on the behavior patterns change after removing macroeconomic factors. The results show that: (1) changes in either the demographic structure or behavior patterns due to higher education explain 63.41% or 36.59%, respectively, of the average marriage age delay; (2) the macro factors would delay the age of first marriage; (3) after controlling for demographic structure and macro factors, 3 years or more of higher education would only delay the choosing behavior of entering the first marriage by 0.84 years. Thus, we concluded that higher education does not completely squeeze the time of marriage, and the expansion of college enrollment could improve social and economic benefits.

## 1. Introduction

In the Fifth Plenary Session of the 19th Central Committee of the Communist Party of China, the problem of population aging was raised as a topic to be addressed using a national strategy during the process of rapid urbanization. The aging population and declining birth rate have led to a shortage of labor force, thus causing the goal of improved labor productivity to become a matter of urgency (Wu and Liu, 2015). So, the policymakers in China paid more attention to human capital investment. One of the important ways to increase human capital stock was to promote higher education (Wu, 2010). In 1999, China began to implement college enrollment expansion to promote the education level and productivity for high-quality urbanization, which greatly increased the enrollment rate of higher education in China. With the increase in the number of people receiving higher education, however, there was a growing paradox that the spread of higher education will further delay the age at which men and women first get married (Zhu and Zhao, 2019). The trade-off between the accumulation of human resources and aging population growth has forced us to consider whether the popularity of higher education has changed people’s behavior patterns and preferences for choosing the time of marriage, or whether higher education has changed the educated population structure, thus in turn affecting the average age of the first marriage.

The limitations of the existing literature are mainly related to two aspects. First, most literature have failed to distinguish the influence of college enrollment expansion on people’s behavior patterns and the change in population composition (Wang and Shi, 2014; Zhu and Zhao, 2019). Theoretically, the proportion of the population with higher education can reach 100% at most; that is, there is an upper limit on the influence of population composition change on the age of first marriage, and what we need to be concerned with is the degree by which higher education influences people’s behavior patterns in choosing the time of first marriage.^1^ We defined this general divergence in the interpretation of delayed age of the first marriage as the “fallacy of average” with respect to age at first marriage. If the average age of the first marriage under the “fallacy of average” was used as the expression of an individual’s first marriage age (i.e., the individual’s choosing behavior of getting first married), people would misestimate the influence of higher education on changing an individual’s behavior pattern.

**Figure 1:**
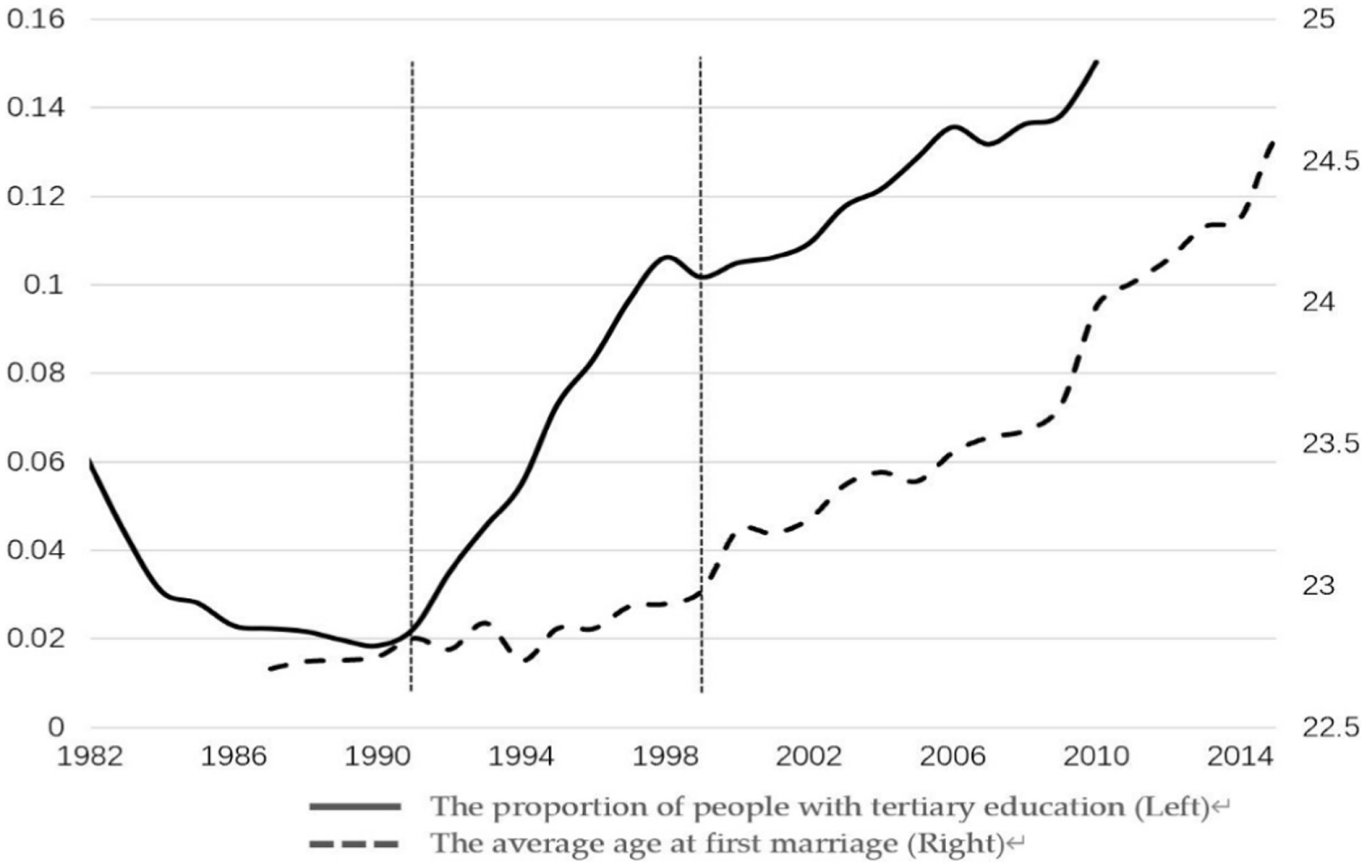
Trends in the proportion of people with tertiary education and the average age at first marriage, 1985–2017. China Population and Employment Statistics Yearbook and 2010 National Population Census.

Second, most existing literature have used “experimental comparison” models, such as the Difference-in-Differences method (DID) or synthetic control method (Xing and Li, 2011; Liu and Liu, 2018). Such models tend to mix the influence of macro variables on the age of first marriage with that of higher education to a certain extent, and the results cannot be used for further prediction.^2^ Figure 1 shows the trends for the average age of the first marriage and the proportion of people with a college education or above in China. As can be seen from the figure, the average age of the first marriage in China switched from a decline to an increasing trend in 1991. However, before the enrollment expansion of colleges and universities in 1999, the proportion of people with college or higher education did not significantly increase. The asynchronization of change in the age of first marriage and the implementation of the college enrollment expansion policy implied that we had to re-examine the influence of higher education on the age of first marriage.

Based on the above literature review and considering the limitations in the previous literature, this paper hypothesized that changes in not only the behavior patterns but also the demographic structure of the population received higher education would delay the average age at first marriage, and the macroeconomic factors would also influence the average age at first marriage. As mentioned above, we care more about the impacts on behavior patterns change, so, we need to further test the pure effect of achieving higher education on the change in an individual’s choosing behavior after controlling for demographic structure change and macro factors to evaluate the trade-off between the accumulation of human resources and aging population growth of receiving higher education.

The contributions of this paper are as follows: First, the conclusions of the previous literature tend to overestimate the delayed age of first marriage caused by higher education due to the aforementioned errors. This paper decomposed this influence into extensive and intensive margins and corrected the bias in the conclusions of existing literature.^3^ Second, we confirmed the influence of macroeconomic factors on the age at first marriage. Third, the present paper re-estimated the pure impact of higher education on the behavior patterns change of first marriage. If the delay of individual first marriage age is an opportunity cost of attaining higher education, then the results in this paper measured this cost and provided strong empirical evidence for developing countries like China, which need to accumulate human capital and solve the problem of population aging, to continue promoting college enrollment expansion. In the terms of methodology innovation, the duration model is used to provide more details on the dynamic process of behavior patterns change at the age of the first marriage. The duration model pays more attention to the dynamic changes of the event occurrence process and can effectively deal with the data merging problem (Blossfeld et al., 2019).

## 2. Literature review

The theoretical study of family economics began with Becker (1973, 1974) assumed that each rational person provided labor to the “family” and the “market” and obtained benefits, pursuing utility maximization based on their preferences. Then, in a perfectly competitive labor market, people would tend to choose “family” only when the total production of both men and women forming a family was greater than the sum of their individual products, and the utility could be transferred between husband and wife; otherwise, they would remain single. Since income was an increasing function of education, increasing the level of education would undoubtedly increase people’s utility level in the “market,” which in turn increases the opportunity cost of choosing a “family” and leads to a delay in the age of first marriage (Sewell and Shah, 1967; Sewell et al., 1970; Wen, 2007; Yang and Huang, 2010). Further, based on the theory of gender division of labor proposed by Parsons (1949) and Becker (1981), on the one hand, believed that the sex ratio (number of males/number of females) was the key factor determining marriage composition and income, and the change of the sex ratio will affect the marriage rate and total family utility. On the other hand, he pointed out that women had a comparative advantage in family labor, and the improvement of education level would reduce the relative benefits of traditional thought that women should take charge of the family, so the impact on the age of first marriage of women would be higher than that of men.

Subsequent empirical research analyzed the impact of higher education on the age of first marriage in detail, and the resulting viewpoints can be mainly divided into three categories: first, the improvement of women’s education level and economic status. When women are more educated and thus can earn higher incomes in the market, the increased opportunity cost of returning to the family would lead women to reduce their willingness to marry, therefore delaying the age of first marriage (Kaufmann et al., 2013; Requena and Salazar, 2014; Chen, 2015; Wu and Liu, 2015; Hahn et al., 2018). The second viewpoint is concerned with the time allocation decision between academia and marriage (Raymo, 2003; Yang and Zhang, 2018; Marphatia et al., 2020). When the time available for consumption was given, education would inevitably squeeze marriage time (Addo et al., 2019). Third, the expansion of college enrollment has led to an imbalance in the gender ratio (Cohen and Pepin, 2018; Lan et al., 2019). The decline in the sex ratio would reduce the market demand for market-replaceable “household products” provided by traditional women, thereby increasing the labor force participation rate of women in the market and delaying the age of first marriage (Chiappori et al., 2018). The gender gap in educational attainment has been gradually narrowing, and there is even a phenomenon of women surpassing men (Wu, 2012). To sum up, higher education can delay the average age of the first marriage due to two aspects: the change in behavior patterns of different types related to individuals’ age at first marriage, and the demographic changes associated with overall educational level.

In terms of how much higher education will delay the average age of the first marriage, most of the existing literature focuses on the comprehensive impact of higher education on the age of first marriage, and the conclusions are not the same. According to the study by Zhu and Zhao (2019), each additional year of higher education will delay the average age of the first marriage by 1.5 years. However, according to Ge and Huang (2020), the expansion of college enrollment will delay the age of first marriage and first childbearing by 1.28 and 1.63 years, respectively. There are also a few studies focusing on the influence of demographic change on the marriage rate. Liu and Liu (2018) analyzed the synchronization of the change between demographic structure and marriage rate and concluded that the expansion of college enrollment has contributed to an increase in marriage rates in China; In terms of the average age at first marriage, Liu (2016) compared the influence of the change of population composition in each education level on the average age at first marriage by using the median age at first marriage of the population of all ages with two census data. The results suggested that the demographic changes caused by the expansion of university enrollment account for 78 and 50% of the delayed age of first marriage for men and women aged 23 at the time of the Sixth National Census.

According to the aforementioned reasons, what we should focus more on was the impact of higher education on the change of choosing behavior of getting first married. However, the existing papers neglected the demographic structure change due to higher education and did not extract this pure effect on the behavior patterns change. In addition, macroeconomic factors may also influence the average age of the first marriage. This paper is devoted to testing the explanatory ability of the demographic structure and behavior patterns change due to higher education to the average age of the first marriage, then evaluate the pure impact on choosing behavior of getting first married eliminating macroeconomic factors.

## 3. Data and methodology

### 3.1. Data description

Using data from the China General Social Survey (CGSS) for the year 2017, this paper examined how higher education affects the change of age at first marriage for men and women. Led by the Renmin University of China, the CGSS has sampled more than 10,000 households across the country since 2003. The sample covers 12,582 households in 28 provincial-level administrative regions (except special administrative regions, Taiwan and Hainan provinces, Xinjiang Uygur and Tibet Autonomous Regions),^4^ including 8,043 urban households and 4,539 rural households. The gender ratio of respondents was relatively balanced, which was in line with the Sixth National Population Census Survey. At the same time, to clarify the place where residents attained education, we took the urban and rural residences of individual samples at the age of 14 as the reference value for their permanent residence. In addition, since the economic environment influences the decision-making of older residents is quite different from today, given that younger residents may not have completed their studies and have a low proportion of married people, only residents born between 1945 and 1984 were selected, and they were divided into three generations. The final effective sample size was 8,292.^5^

The following variables were selected for exploring the influence of higher education on the age of first marriage:

1. Main explanatory variables

The core explanatory variable in this paper was whether or not higher education was attained, with a value of 1 and 0, respectively. Regarding the birth cohort, the first, second, and third generation referred to the residents born in the year 1945–1959, 1960–1969, and 1970–1984, respectively, and the value assigned to it was 0, 1, and 2, respectively. Gender was a binary variable with a value of 0 for males and 1 for females. Due to the impact of China’s urban–rural development differences on education development, the urban–rural gap is a core issue impacting the education gap (Pei, 2006; Shen et al., 2013); to control this impact, we selected the residence of the respondents at the age of 14 as the indicator for their permanent residence type, where the value for rural areas was 0, and the value for urban areas was 1.

1. Micro-control variables

There are three micro-control variables in the present regression: the parent’s level of education, the number of siblings, and the maximum age difference between the parents and their children. The two variables of parents’ level of education and number of siblings have significant effects on an individual’s first marriage age and were thus added to the regression (Wang and Wu, 2013). The values of 0, 1, 2, and 3 correspond to the level of education of the respondent’s parents as represented by unknown education level, primary education level or below, secondary education level, and tertiary education level, respectively. As for the maximum age difference between parents and their children, Zhang and Tan (2021) argued that the greater the age difference between the parents and their children, the more serious the phenomenon that the parents would pressure you to get married in China.

1. Macro-control variables

Four macro-control variables were added in this paper: the moving average growth rate of real GDP of the respondents aged 20 and 29, unemployment rate, degree of economic openness, and marriage rate of the population aged 20–25 when the respondents are aged 25.

The moving average growth rate of real GDP was calculated from nominal GDP and GDP deflator taking 1978 as the base year. Considering that China had not completed the economic marketization process before the year 1978 and that the inflation rate was relatively low, nominal GDP, instead of real GDP, was used to calculate the growth rate before 1978 (Li et al., 2018). The unemployment rate was the registered urban unemployment rate calculated by the National Bureau of Statistics when the respondents were aged 25. The degree of economic openness was measured by the degree of dependence on foreign trade, which was calculated by dividing import and export data collected by the National Bureau of Statistics by the gross domestic product. For the last macro variable, the marriage rate was calculated as a percentage of the population aged 20–25 who got married each year between 1970 and 2000 based on data from a sample of more than 10,000,000 people in China collected from the year 2000 IPUMS international dataset. The increase in the proportion of married people in the same age group would place pressure on unmarried people to get married, which would lead to an increase in the probability of unmarried people getting married (Wang and Wu, 2013); therefore, this paper used this variable as a measure of exogenous “peer pressure.”

Table 1 presents descriptive statistics of the data. It can be seen from Table 1 that the proportion of people with higher education gradually increases over time and the growth rate continually rises, but the average age of the first marriage in the second generation was earlier than that of the other two generations. Liu and Liu (2018) argued that, compared with the Marriage Law in 1950, the policy of late marriage and childbearing implemented in 1973 raised the age of residents at first marriage by 5 years, while the New Marriage Law in 1980 only delayed the age of first marriage by 2 years and advanced the policy of late marriage and childbearing by 3 years. Therefore, the age of first marriage of second-generation residents has dropped.^6^ The changing trend of the age at first marriage of residents in different generations is basically the same as that in the Sixth National Population Census Survey, which indicates that the chosen sample is generally representative. In addition, the mean values of residence at age 14 were close to 0 as time passed by, which may indicate that the expansion of higher education accelerate the process of urbanization.

**Table 1:** Descriptive statistics.

Variables	Birth cohort		
	The 1st generation	The 2nd generation	The 3rd generation
The average age of first	24.16	23.49	24.45
Marriage	(4.173)	(3.986)	(3.678)
Whether to attain the	0.07	0.11	0.26
Higher education (yes = 1)	(0.248)	(0.313)	(0.438)
Gender(Female = 1)	0.51	0.50	0.46
	(0.500)	(0.500)	(0.499)
Residence at age 14	0.30	0.27	0.25
(Rural = 1)	(0.458)	(0.430)	(0.442)
Parent’s level of	1.09	1.21	1.45
Education	(0.435)	(0.543)	(0.610)
Number of siblings	1.23	1.13	0.68
	(2.580)	(1.966)	(1.337)
Maximum age difference	29.29	30.51	29.06
	(7.582)	(6.872)	(6.116)
Moving average growth	0.07	0.10	0.10
Rate of real GDP	(0.012)	(0.004)	(0.004)
Degree of economic openness	0.05	0.14	0.24
	(0.016)	(0.036)	(0.065)
Unemployment rate	0.04	0.02	0.03
	(0.006)	(0.003)	(0.005)
Marriage rate of the	0.11	0.12	0.10
Population aged 20–25	(0.022)	(0.013)	(0.016)
No. of obs.	3,148	2,330	2,814

2017 CGSS (the numbers in brackets are standard deviations).

**Figure 2:**
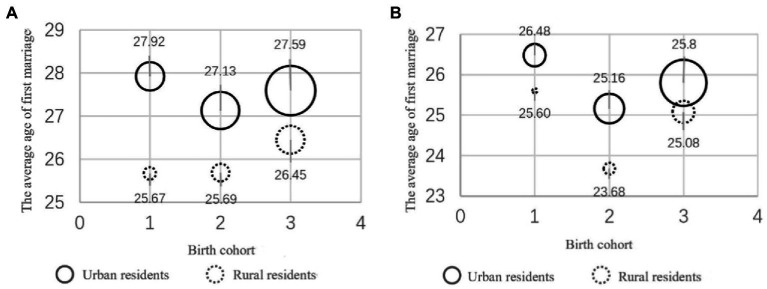
Population distribution by generation, urban and rural, and the average age distribution at first marriage. **(A)** Male residents. **(B)** Female residents. The numbers 1, 2, and 3 on the horizontal axis represent the first, second, and third generations, respectively. 2. The size of the bubble corresponds to the proportion of the population with higher education.

Furthermore, Figure 2 shows the trend of how demographic structure changes with higher education and the age of first marriage according to gender, birth generation, and urban and rural areas. The size of the bubble corresponds to the proportion of the population with higher education in the indicated group. As can be seen from Figure 2, after the sample residents were classified, the average age of the first marriage for rural males rose from the first to the third generation, which was not consistent with the overall trend (i.e., the average age of the first marriage was lowest for the second generation). If we only focused on the overall average age of first marriage, the proportion of residents born in the first generation with higher education in this sample was 6.61%, and that of the second generation was 11.03%. After taking into account the actual age of first marriage of all groups, the overall average age of first marriage of the first generation was 25.00 years old, higher than the value of 24.71 years old for the second generation. The average age at first marriage thus does not accurately reflect the changes in the age at first marriage of different types of residents (in this case, rural males), which fully demonstrates the potential impact of changes in the composition of educated groups on the average age at first marriage.

### 3.2. Methodology specification

The asynchrony in the expansion of higher education and the transition in the age at first marriage in Figure 1 made us realize that the factors affecting the average age at first marriage are not limited to higher education, so we needed to control for other variables in the regression, and we, therefore, removed the effect of higher education on the average age at first marriage. This paper uses duration analysis to explore the differences in age at first marriage among urban and rural residents of different generations, education levels, and gender, and this model is used to eliminate the influence of micro factors on the average age at first marriage. Duration analysis, also known as survival analysis or life-table analysis, is a statistical method to investigate the time it takes for an object to change from one state to another, and it is widely used in biology, demography, and economics.

Compared with the DID or synthetic control models, the advantage of duration analysis is that it provides more detailed information and can effectively deal with data merging while paying attention to the dynamic changes in events. Sample merging is very common in the analysis of the age of first marriage. For example, the data used in this study included 1,316 unmarried individuals, and the fact that they did not enter their first marriage at the time of investigation did not mean that they will not enter a marriage state in the future. The processing of merging data in the duration analysis thus makes the results more reliable.

In this paper, entering the first marriage was set as the event occurrence. The initial time was set as 16 years old, and the cases who had not married over 33 years old were censused to the right. Within the age group that this article focused on (i.e., 16–33 years old), the risk rate at which individuals got married will gradually increase (Wang and Wu, 2013). The Gompertz model in duration analysis is suitable when the risk increases or decreases exponentially with time.^7^ Therefore, we chose the Gompertz model for regression. The equations for this model are:


(1) h(t)=λexp(γt)

(2) $$\begin{array}{c} S\left(t\right)=\exp\left\{-\lambda \gamma^{-1}\left(e^{\gamma t}-1\right)\right\} \end{array}$$

where h(t) is the risk function of the Gompertz model, S(t) is the corresponding survival function, γ is the parameter to be estimated, t is the time experienced before the event occurs (Michael and Lee, 1994), and we control individual heterogeneity in regression, making regression results more reliable.

After controlling the possible influence of micro and macro factors, we constructed the following formula to decompose the influence of higher education on the average age of the first marriage in terms of extensive margin and intensive margin into changes in population composition and behavior patterns:


(3) $$\begin{array}{c} {\bar{age}}_{y}=\overset{n}{\underset{g=1}{\sum }}\left(age_{y}^{g}\times share_{y}^{g}\right) \end{array}$$

where ${\bar{age}}_{y}$ represents the average age of the first marriage in year y; $age_{y}^{g}$ represents the average age of the first marriage of the population of type g (such as men with higher education, etc.) in this year and thus the change in the behavior patterns of the population; $share_{y}^{g}$ represents the proportion of different groups in the total population of the year, which represents the change in population composition. The change in average age at first marriage can then be decomposed into:


(4) $$\begin{array}{c} {\bar{age}}_{y}-{\bar{age}}_{y^{\prime }}=\overset{n}{\underset{g=1}{\sum }}\left(age_{y}^{g}\times share_{y}^{g}\right)-\overset{n}{\underset{g=1}{\sum }}\left(age_{y^{\prime }}^{g}\times share_{y^{\prime }}^{g}\right) \end{array}$$

where


(5) $$\begin{array}{c} age_{y^{\prime }}^{g}=age_{y}^{g}+\Delta age^{g} \end{array}$$

(6) $$\begin{array}{c} share_{y^{\prime }}^{g}=share_{y}^{g}+\Delta share^{g} \end{array}$$

so


(7) $$\begin{array}{l} {\bar{age}}_{y}-{\bar{age}}_{y^{\prime }}=\overset{n}{\underset{g=1}{\sum }}\left(\Delta age^{g}\times share_{y}^{g}\right)+\overset{n}{\underset{g=1}{\sum }}\left(age_{y}^{g}\times \Delta share^{g}\right)\\ +\Delta age^{g}\times \Delta share^{g} \end{array}$$

It can be seen from Equation (7) that the change in the average age of the first marriage was decomposed into the unchanged population composition multiplied by the change of behavior pattern, the unchanged behavior patterns multiplied by the change of population composition, and the cross-product term when both behavior patterns and population composition change. In order to avoid the influence of different calculation order on the result, we evenly distributed the influence of the cross-product term to the factors of population composition change and behavior patterns.

## 4. Empirical results and analysis

### 4.1. Main regression results

Table 2 reports the main results of the duration model. Model 1 only contains the main explanatory variables and is a baseline model for the comparison of the follow-up models. As can be seen from the regression results in the second column of Table 2, the coefficients of the variables on whether higher education was attained were significantly negative, and the coefficients of the other main explanatory variables were significant at the level of 1%. Before controlling for any micro factors, the age at which individuals with higher education first entered into marriage was 1.70 years later than those without higher education, while the first marriage age of women was about 1.27 years earlier than that of men; the urban residents were 1.58 years later than rural residents to get married for the first time, which were in line with the results of Wu (2012) and Shen et al. (2013). For different generations, the age of the first marriage for the second generation was significantly earlier than that of the first generation, which is consistent with descriptive statistical analysis. However, the coefficient of the third generation was not significant, which inspired us to go deeper with the analysis.

**Table 2:** Analysis of influencing factors of the average age of the first marriage.

Variables	Model 1	Model 2	Model 3	Model 4	Model 5
Whether to attain the	−1.703^***^	−1.518^***^	−1.380^***^	−1.291^**^	−0.842^**^
Higher education (yes = 1)	(0.112)	(0.126)	(0.323)	(0.42)	(0.333)
The 2nd generation	0.470^***^	0.692^***^	0.711^***^	0.148	0.314
	(0.088)	(0.113)	(0.174)	(0.32)	(0.274)
The 3rd generation	−0.053	−0.236^**^	0.137	−0.258	−0.006
	(0.086)	(0.108)	(0.171)	(0.34)	(0.297)
Gender	1.298^***^	1.272^***^	1.081^***^	0.838^***^	1.094^***^
(female = 1)	(0.073)	(0.087)	(0.184)	(0.235)	(0.200)
Residence at age 14	−1.581^***^	−1.409^***^	−1.427^***^	−1.543^***^	−1.829^***^
(Rural = 1)	(0.094)	(0.108)	(0.108)	(0.133)	(0.121)
The 2nd gen×			0.227	0.0251	−0.135
Higher education			(0.452)	(0.535)	(0.422)
The 3rd gen×			−0.318	−0.315	−0.360
Higher education			(0.369)	(0.537)	(0.478)
The 2nd gen × female			−0.078	0.218	0.006
			(0.230)	(0.279)	(0.239)
The 3rd gen × female			0.386^*^	0.704^*^	0.352
			(0.227)	(0.316)	(0.281)
Higher education			0.259	0.0560	−0.390
×Female			(0.501)	(0.540)	(0.473)
The 2nd gen × higher			−0.201	−0.0358	0.763
Education × female			(0.703)	(0.753)	(0.621)
The 3rd gen × higher			−0.248	0.260	1.017
Education × female			(0.566)	(0.702)	(0.660)

Micro variables	N	Y	Y	Y	Y
Macro variables	N	N	N	Y	Y
Gamma	0.868^***^	0.877^***^	0.880^***^	0.927^***^	0.902^***^
	(0.019)	(0.024)	(0.024)	(28.59)	(0.028)
Lntheta	1.097^***^	1.083^***^	1.084^***^	1.079^***^	0.963^***^
	(0.038)	(0.046)	(0.046)	(18.56)	(0.057)
No. of obs.	5,721	5,721	5,721	3,593	3,593

1. ^*^, ^**^, and ^***^ are significant at 0.1, 0.05, and 0.01 levels, respectively. 2. The standard errors are reported in brackets. 3. Micro variables include the parent’s level of education, the number of siblings, and the maximum age difference between the parents and their children. Macro variables include the moving average growth rate of real GDP of the respondents aged 20 and 29, unemployment rate, degree of economic openness, and marriage rate of the population aged 20–25 when the respondents are aged 25. 4. The significance of the gamma coefficient indicates that exponential regression should be rejected and Gompertz regression should be selected. The significance of the lntheta coefficient indicates acceptance of the assumption of heterogeneity.

Model 2 added micro-control variables on the basis of Model 1. At this time, the coefficient of the second generation increased compared with that of Model 1, and the coefficient of the third generation was significant at the 5% level. At the same time, the influence of gender and residence at the age of 14 on the average age at first marriage decreased slightly, indicating that micro-control variables had a significant impact on the average age at first marriage.

Model 3 further added the interaction terms of birth cohort, gender, and whether higher education was attained. The purpose was to investigate whether the behavior patterns of individuals born in different generations on age at first marriage would simply change over time, which would bias the effect of higher education on the age at first marriage. The fourth column in Table 2 shows the regression results of Model 3. As can be seen from rows 6–12, none of these interaction term coefficients was significant. Based on the coefficients for the interaction of birth cohort and higher education, we can conclude that compared with the first generation of residents, the impact of higher education on the age of first marriage did not become increase nor decrease in the second and third generations, and there was no significant difference in the age of first marriage between three generations. That is, the individual behavior patterns did not simply change with the generations. In other words, the results revealed that if individuals’ gender and education level were the same but they were born in different generations, there would not be significant differences in the time they entered their first marriage. Finally, compared to Model 2, the age at first marriage of highly educated individuals decreased further, and was only 1.38 years later than those of people who have not received higher education.

Comparing Models 1–3, it can be seen that the coefficient of the third-generation variable varies greatly, and the reason may be that the generation effect was the aggregate result of multiple influencing factors. We assumed that the sum of the generation effects is 0. After controlling for factors such as gender, education level, and urban and rural areas, the effects of other influencing factors were prominent, resulting in a large change in the coefficient of the third generation compared with the first generation in Model 2, whose age at first marriage was significantly delayed. This effect disappeared after further controlling for the cross-section term.

**Figure 3:**
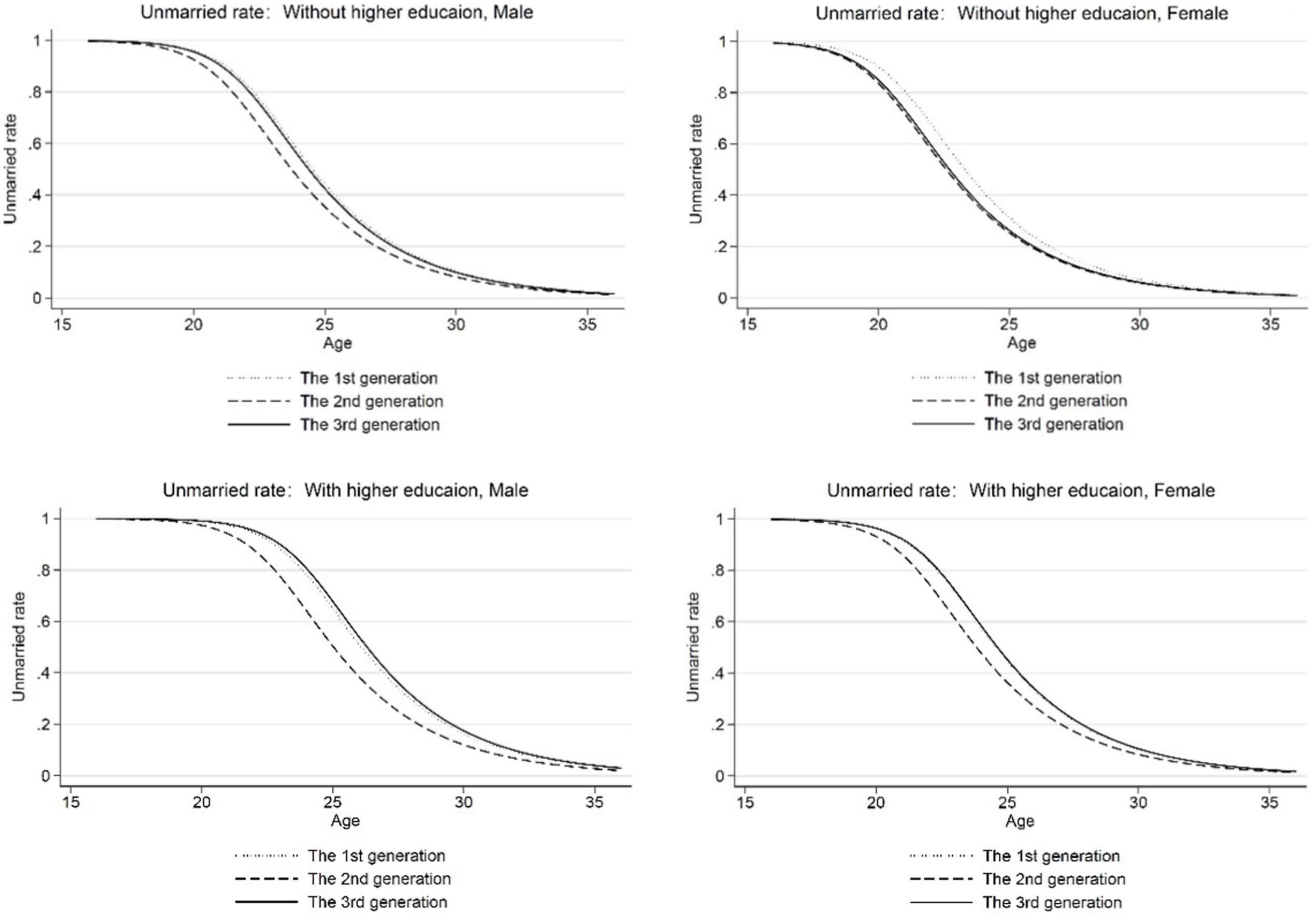
Age—unmarried rate (controlling for micro variables). Micro variables include the parent’s level of education, the number of siblings, and the maximum age difference between the parents and their children.

Figure 3 further shows the regression results of Model 3. It can be seen from the figure that after controlling for micro variables, the preference of residents born in the first and third generations for the behavior of choosing to enter marriage did not change significantly. The residents born in the second generation had an earlier marriage age than the other two generations. From the descriptive statistics, it can be seen that the proportion of the population with higher education in the second generation is higher than that of the first generation and less than the third generation, and the coefficient of whether to attain higher education was still significantly negative. Hence, the reason why second-generation residents marry later may be related to macroeconomic factors. As mentioned earlier, Becker (1981) stated that the relative advantages of individuals between “market” and “family” will determine whether individuals enter marriage, so the impact of “market” conditions on the age of first marriage cannot be ignored. Therefore, we would further strip macroeconomic factors from generational effects to explore the effect of higher education on the average age at first marriage.

**Figure 4:**
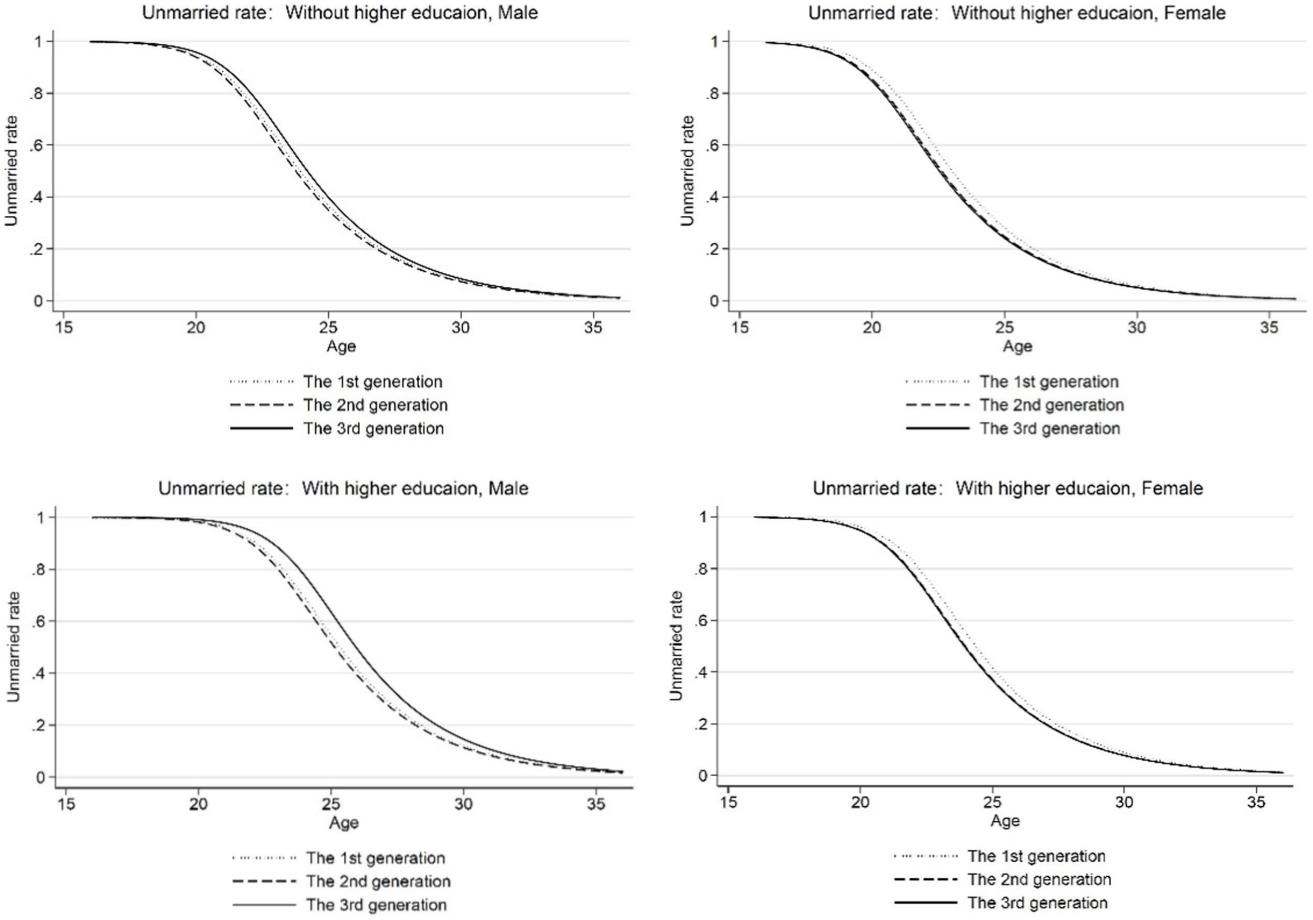
Age—unmarried rate (controlling for micro and macro variables). Micro variables include the parent’s level of education, the number of siblings, and the maximum age difference between the parents and their children. Macro variables include the moving average growth rate of real GDP of the respondents aged 20 and 29, the unemployment rate, degree of economic openness, and marriage rate of the population aged 20–25 when the respondents are aged 25.

Model 4 in Table 2 shows the results after controlling for macro and micro variables. At this time, the coefficients of the second and third generations are not significant, indicating that after eliminating the influence of macro factors, the first-married age was not significantly different between generations. Figure 4 reports the regression results of Model 4. By comparing Figures 3, 4, it can be seen that after controlling for the macro influencing factors, the age at first marriage of different generations was more consistent than before, and particularly the age at first marriage of second-generation residents is significantly delayed. Specifically, the age—unmarried probability curve of residents born in 1960–1969 in Figure 4 is closer to the age—unmarried probability curve of residents born in the first and third generations than that in Figure 3, and this comprehensively demonstrates that macro-influencing factors do affect individual behavior patterns related to first marriage. Compared with the previous model, the variable coefficient of whether or not to attain higher education in Model 4 further decreased (row 3, column 2 in Table 3), indicating that after controlling for macro and micro variables, attaining higher education would only delay the average age of the first marriage by 1.29 years, which is consistent with the conclusion of Ge and Huang (2020).

**Table 3:** The robustness tests.

Variables	Model 3	Using Model 4 sample size regress Model 3
Whether to attain the	−1.380^***^	−1.335^***^
Higher education (yes = 1)	(0.323)	(0.422)
The 2nd generation	0.711^***^	0.877^***^
	(0.174)	(0.220)
The 3rd generation	0.137	0.425
	(0.171)	(0.247)
Gender(Female = 1)	1.081^***^	0.817^***^
	(0.184)	(0.238)
Residence at age 14	−1.427^***^	−1.570^***^
	(0.108)	(0.134)
The 2nd gen × attaining	0.227	0.088
Higher education	(0.452)	(0.532)
The 3rd gen × attaining	−0.318	−0.246
Higher education	(0.369)	(0.538)
The 2nd gen × female	−0.078	0.241
	(0.230)	(0.282)
The 3rd gen × female	0.386^*^	0.727^*^
	(0.227)	(0.319)
Attaining higher	0.259	0.095
Education × female	(0.501)	(0.543)
The 2nd gen × attaining	−0.201	0.260
Higher education × female	(0.703)	(0.750)
The 3rd gen × attaining	−0.248	0.193
Higher education × female	(0.566)	(0.706)
Micro variables	Y	Y
Macro variables	N	N
Gamma	0.880^***^	0.929^***^
	(0.024)	(0.033)
lntheta	1.084^***^	1.086^***^
	(0.046)	(0.059)
No. of obs.	5,721	3,593

1. ^*^, ^**^, and ^***^ are significant at 0.1, 0.05, and 0.01 levels, respectively. 2. The standard errors are reported in brackets. 3. Micro variables include the parent’s level of education, the number of siblings, and the maximum age difference between the parents and their children. Macro variables include the moving average growth rate of real GDP of the respondents aged 20 and 29, the unemployment rate, degree of economic openness, and marriage rate of the population aged 20–25 when the respondents are aged 25. 4. The significance of the Gamma coefficient indicates that exponential regression should be rejected and Gompertz regression should be selected. The significance of lntheta coefficient indicates acceptance of the assumption of heterogeneity.

### 4.2. Extensive and intensive margins

Based on the above regression analysis, we can conclude that the impact of higher education on the first-married age was not so severe compared to the existing papers (Zhu and Zhao, 2019), we further used Equation (7) to explore the impact of demographic structure and behavior pattern changes caused by higher education on the average age of the first marriage. Since the average age of the first marriage for the first and third generations of residents barely changed, this paper only selected the second and third generations of residents to test the explanatory ability of the influence of higher education on the age of first marriage in the extensive margin and intensive margin. We summed the weights of the second and third generations of residents by generation, then adjusted the total weight according to the unit weight and divided it by the original weight to get the weight of the individual in the current generation of residents. Further, we multiplied the duration data of different types of individuals by the adjusted weights and took second-generation residents as the reference group. By multiplying the dynamic change data of second-generation residents by the population weight composition of third-generation residents, we explored the influence of population composition change on the average age at first marriage. We then controlled the population weight of second-generation residents and multiplied the duration data of third-generation residents at first marriage to discuss the effect of behavior patterns change on the age at first marriage.

**Figure 5:**
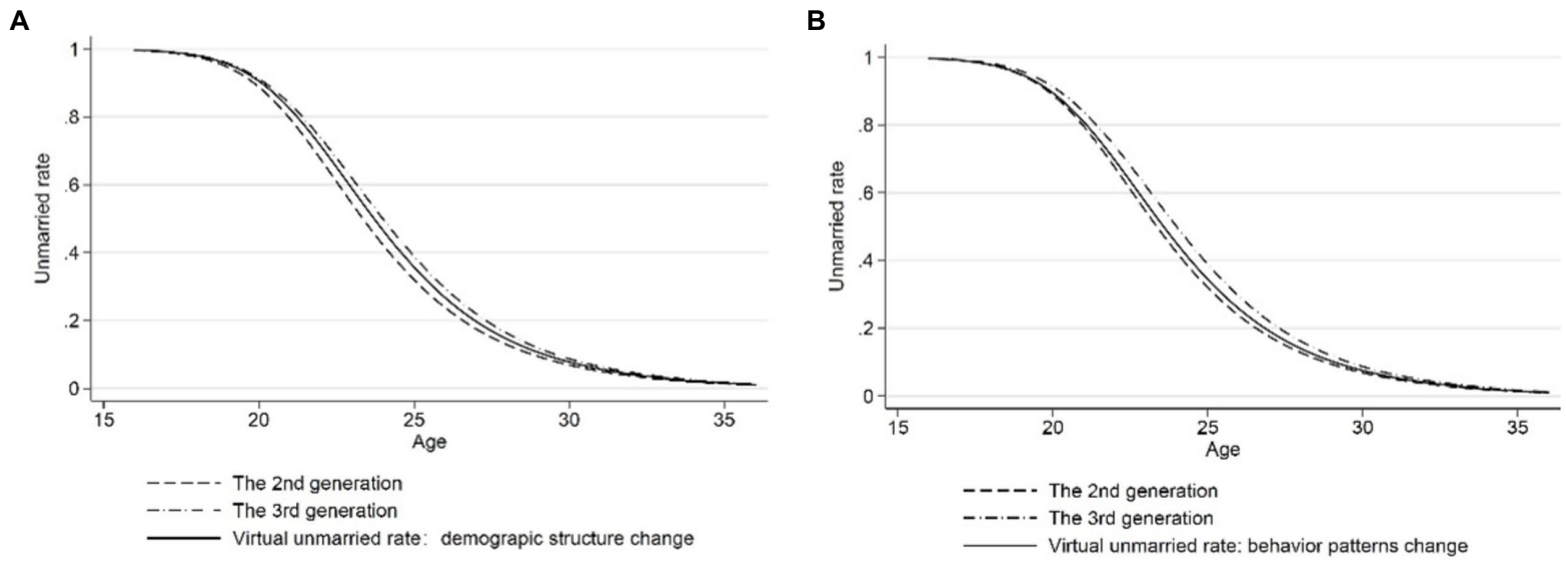
Age—unmarried rate (controlling for demographic structure or behavior patterns change). **(A)** Age—unmarried rate after controlling for demographic structure change. **(B)** Age—unmarried rate after controlling for behavior patterns change.

Through the calculation of Equation (7), it can be concluded that the explanatory ability of changes in either demographic structure or behavior patterns caused by higher education to delay the age of first marriage is 63.41% or 36.59%, respectively. Figure 5 shows the analysis of the influence due to changes in either population composition or behavior patterns caused by higher education on the age of first marriage. Taking second-generation residents as the reference group, the solid line in Figure 5A shows the individual’s choice of age at first marriage when the behavior pattern of second-generation residents is controlled and the demographic structure was changed to third-generation. The solid line in Figure 5B shows the marriageable age when controlling the demographic structure of second-generation residents and changing the behavior pattern to third-generation residents. According to the spacing between the virtual unmarried rate and the unmarried rate of second-generation residents, it can be seen that the impact of demographic structure change caused by higher education on the age of first marriage was greater than that of delaying first marriage caused by behavior composition change.

Model 4 in Table 2 shows that attaining higher education will delay the average age of the first marriage by 1.29 years before controlling for changes in the demographic structure, and the increase in the proportion of the population with higher education could explain 63.41% of the delay in the age of first marriage, which was around twice of the explanatory ability of behavior patterns change. Therefore, we controlled for population composition on the basis of Model 4, and the regression results are shown in Model 5 in Table 2. According to Model 5, after controlling demographic structure and macro and micro factors, attaining higher education only delays people’s behavioral choice regarding the age of first marriage by 0.84 years, which was far shorter than the time required by attaining higher education (usually 3 years or more).

### 4.3. Robustness tests

The first test of robustness addressed changes in sample size. Due to the absence of some macro variables, the sample size of Model 4 is only 3,593. In order to avoid deviation in the regression results of the models caused by changes in sample size, we used this sample to re-estimate Model 3, and the estimation results are shown in the third column of Table 3. Compared with Model 3, the coefficients of Model 3 when re-estimated using the samples of Model 4 do not change much. The reason for the change may be due to insufficient sample size. The sign and significance of each coefficient did not change, so it can be considered that the regression result is relatively robust and reliable.

**Figure 6:**
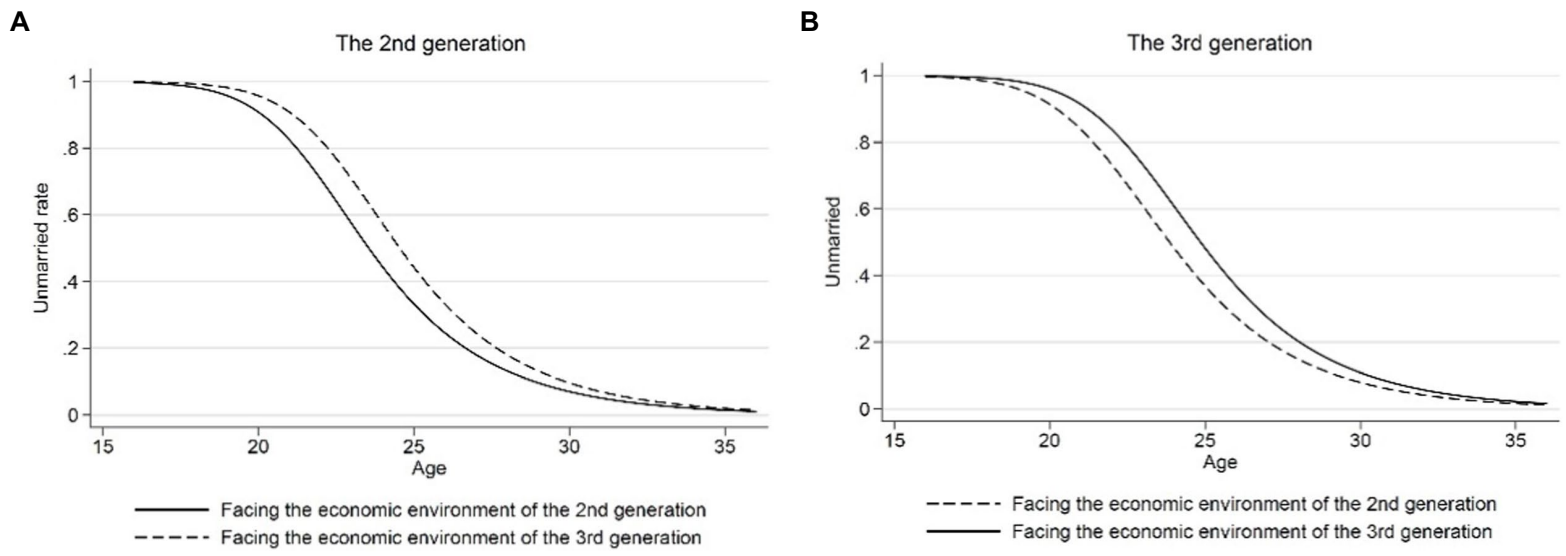
The influence of macroeconomic factors on the average age at first marriage. **(A)** The 2nd generation. **(B)** The 3rd generation. Macro variables include the moving average growth rate of real GDP of the respondents aged 20 and 29, the unemployment rate, degree of economic openness, and marriage rate of the population aged 20–25 when the respondents are aged 25.

The second robustness test addressed the influence of macroeconomic factors on the age of first marriage of second-generation residents. As can be seen from the above figures, the age of first marriage of residents born in the second generation was earlier than that of those born in the first and third generations before controlling for macroeconomic variables. However, such generational characteristics were no longer significant after controlling for macroeconomic factors. In order to further test our conclusion that the macroeconomic environment leads to generational differences in the average age at first marriage, we selected the second-and third-generation samples to test the impact of macro variables on the age at first marriage and examined whether the residents would choose different age of first marriage when born in different generations and thus faced with a different economic environment. Figure 6 reports the results of analyzing this problem. Figures 6A,B show the age of first marriage for residents born in the second and third generations facing their own economic environment and the economic environment of another generation. It can be seen from the figure that macro factors had a significant impact on the age of first marriage, and no matter the generation or gender of residents, when they are in the economic environment of the second generation, the time they choose to enter first marriage will be delayed, which serves as theoretical support for explaining the aforementioned phenomenon.

## 5. Discussion and conclusion

Although there has been much discussion on the effect of higher education on the age at first marriage in the existing literature, most reports equate the total effect of higher education to the effect of individual behavior patterns on the average age at first marriage, thus ignoring the potential impact of demographic structure changes caused by the expansion in higher education. Moreover, most of the existing literature used controlled experimental models such as the DID method, in which it is easy to confuse the impact of macro-influencing factors with that of the impact of higher education and thus overestimate the impact of higher education on postponing the age of first marriage. By using the CGSS data for the year 2017, this paper applied a duration model to analyze the explanatory ability of demographic structure and behavior patterns changes in higher education on delaying the age at first marriage, and empirically studied the significant impact of macroeconomic factors on the average age at first marriage. After controlling for demographic structure and macro-and micro-influencing factors, we explored to what extent higher education changes individuals’ behavior patterns to enter the first marriage.

First, the conclusion that attaining higher education will delay the average age of the first marriage has been recognized by most scholars (Liu, 2016; Zhu and Zhao, 2019). Most of the literature on this issue, however, has only focused on the total effect of higher education on the average age at first marriage, and mistakenly concluded or presumed that higher education changes people’s behavior in choosing when to enter marriage. We believe that the reason why the average age of the first marriage in China has been delayed year by year is related to both the composition of the higher-educated population and the change in their behavior patterns. Therefore, after using the duration model to eliminate the relevant macro and micro influencing factors, we used the duration dynamic data to decompose the impact of higher education and then explored the effect of higher education on the average age at first marriage in terms of the extensive and intensive margins. The magnitude of effects was 63.41 and 36.59%, respectively, and the change in the demographic structure caused by higher education was the main reason for the delayed age of first marriage.

Second, after controlling for micro factors, the average age at first marriage of residents born in 1960–1969 (i.e., the second generation) was still lower than that of the first and third generations. Therefore, we evaluated the effect of exogenous macro factors on the age at first marriage. The results showed that after controlling for the macro-influencing factors, the age selection behavior of residents at first marriage tended to be consistent, and exogenous macro factors had a significant impact on the age at first marriage. When residents of different generations are faced with the economic environment of the second generation, the age of first marriage was lower than for residents in the economic environment of the third generation. This result shows that the generational differences in the average age at first marriage are not entirely due to differences in education levels, and macro-influencing factors also play a crucial role.

Finally, the proportion of people with higher education levels can theoretically reach up to 100%. Therefore, the impact of demographic change caused by higher education on the average age of the first marriage has an upper limit. So, we paid more attention to the proportion of the population with higher education, i.e., what was the change in the behavior patterns regarding the age of first marriage for the uninitiated population. Based on the above theoretical analysis, after controlling for the demographic structure and macro and micro variables, we calculated that attaining 3 years or more of higher education would delay the average age of the first marriage by 0.84 years, indicating that higher education is not completely responsible for delaying the time to first marriage. The increase in income for individuals in the “market” was not completely offset by the reduction in “household” income. In general, the popularization of higher education will increase social and economic benefits.

The results in this paper carry very important implications for policies for a developing country such as China, which is in the rapid process of urbanization and faced with an aging population and, therefore, an urgent need to increase labor productivity. In an average sense, higher education still improved personal utility even after being partially offset by the loss of “family”; in an aggregate sense, the combined effect of the “average fallacy” of age at first marriage and macro-and microeconomic factors have caused researchers to overestimate the impact of higher education on the age at first marriage. Since attaining higher education would improve the utility of individuals, the overall economic benefits of society would inevitably be increased. The conclusions of this paper provide a new perspective for us to understand human capital accumulation, and at the same time affirm that the promotion of higher education would help our country transit from having a labor-intensive economy to becoming a capital-intensive country without being excessively aggravated by the aging population.

While this paper focuses on the influence of higher education on the average age of the first marriage, it still neglects important questions such as the mechanism of how higher education influence an individual’s choice to get first married; or shock of the higher education policy to the marriage market. These questions offer directions for future research.

## Data availability statement

Publicly available datasets were analyzed in this study. This data can be found at: http://cgss.ruc.edu.cn/.

## Author contributions

TL is the primary lead author of the article and did most of the data cleaning, regressions and much of the analyses. After finishing the first draft, YH helps write up the paper and made a lot of contributions to the empirical result analyses. JX modified the text part and contributed much to the revision and submission of the manuscript. All authors contributed to the article and approved the submitted version.

## Funding

This research was supported by “the Fundamental Research Funds for the Central Universities” for Scientific Research Innovation Project of China University of Political Science and Law (grant ID: 22ZFQ79001), and “the Program for Young Innovative Research Team in China University of Political Science and Law” (grant ID: 20CXTD10).

## Conflict of interest

The authors declare that the research was conducted in the absence of any commercial or financial relationships that could be construed as a potential conflict of interest.

## Publisher’s note

All claims expressed in this article are solely those of the authors and do not necessarily represent those of their affiliated organizations, or those of the publisher, the editors and the reviewers. Any product that may be evaluated in this article, or claim that may be made by its manufacturer, is not guaranteed or endorsed by the publisher.

## Footnotes

^1^For example, assume that 80% of the population in the first generation did not achieve higher education and 20% of that achieved it, with the average age of the first marriage being 19 and 24, respectively; the proportion of the second generation without higher education is 20% while other 80% achieved it, the average age of the first marriage is 18 and 23, respectively, which is younger than the same group in the first generation. However, the average age of the first marriage of the first generation is 20 years old, and that of the second generation is 22  years old, which will be later than that of the first generation.

^2^For example, China formally joined the WTO in 2000, this event happened almost at the same time as the expansion of college enrollment. When evaluating the impact of the expansion of college enrollment on the average age of the first marriage in China, it is easy to confuse the two causes when using DID or other comparison methods, thus in turn overestimating the impact of the expansion of college enrollment on the age of first marriage.

^3^Drawing on the concepts of extensive margin and intensive margin in economics, this paper argues that the impact of higher education on age at first marriage includes both the impact of the extensive margin—that is, the increased absolute number and proportion of people with higher education; and it also includes the effect on the intensive margin—that higher education changes individuals’ behavior patterns which making people with higher education more inclined to delay marriage.

^4^For special administrative regions, Taiwan, and Hainan provinces, the education systems in these areas are very different from those in the mainland, so it is meaningless to include the data in these provinces; For other autonomous regions, China implemented a policy of extra points in the college entrance examination for ethnic minorities, which included most of the people there in these regions. So, we excluded these regions and provinces in the present paper.

^5^The observed values were divided into three generations: those who entered primary school before the Cultural Revolution (born in the years 1945 to 1959), those who entered primary school during the Cultural Revolution (born in the years 1960 to 1969), and those who entered primary school after the Cultural Revolution (born in 1970 and after). Since education and the social and historical events experienced would affect the values of individuals, and thus their marriage decisions, this division depicted the concept of marriage and love among groups born in different eras to a certain extent. Within a generation, it could be approximated that individuals’ notions of marriage and love are consistent.

^6^This macro policy analysis is not yet supported by empirical results, and we will examine the impact of macro policies on the age of first marriage in the fourth section.

^7^According to the significance of parameters and AIC rule, the Gompertz model was selected for fitting. In addition, we also conducted regression using the exponential model, Weibull model, log-normal model, and Cox model, with no significant differences in the results. Interested readers can ask us for the results for other models.

## References

[ref1] Addo F. R. Houle J. N. Sassler S. (2019). The changing nature of the association between student loan debt and marital behavior in young adulthood. J. Fam. Econ. Iss. 40, 86–101. doi: 10.1007/s10834-018-9591-6

[ref2] Becker G. S. (1973). A theory of marriage: part I. J. Polit. Econ. 81, 813–846. doi: 10.2307/1831130

[ref3] Becker G. S. (1974). A theory of marriage: part II. J. Polit. Econ. 82, S11–S26. doi: 10.1002/9780470755648.part2

[ref4] Becker G.S. (1981). A Treatise on the Family. Cambridge: Harvard University Press.

[ref50] Blossfeld H.-P. Golsch K. Rohwer G. (2019). Event History Analysis with Stata. London: Taylor and Francis.

[ref5] Chen J. (2015). The marital returns of education: "learn well" and "marry well" (in Chinese). J. Shanghai Univ. Fin. Econ. 17, 22–34. doi: 10.16538/j.cnki.jsufe.2015.06.003

[ref6] Chiappori P.-A. Dias M. C. Meghir C. (2018). The marriage market, labor supply, and education choice. J. Polit. Econ. 126, S26–S72. doi: 10.1086/698748

[ref7] Cohen P. N. Pepin J. R. (2018). Unequal marriage markets: sex ratios and first marriage among black and white women. Socius 4:237802311879108. doi: 10.1177/2378023118791084

[ref8] Ge R. Huang J. (2020). Does college enrollment expansion affect marriage and childbearing? (in Chinese). China J. Econ. 7, 168–201. doi: 10.16513/j.cnki.cje.20200723.003

[ref9] Hahn Y. Islam A. Nuzhat K. Smyth R. Yang H.-S. (2018). Education, marriage, and fertility: long-term evidence from a female stipend program in Bangladesh. Econ. Dev. Cult. Chang. 66, 383–415. doi: 10.1086/694930

[ref10] Kaufmann K. M. Messner M. Solis A. (2013). Returns to elite higher education in the marriage market: evidence from Chile. SSRN Electron. J. doi: 10.2139/ssrn.2313369

[ref11] Lan J. Fang Y. Wei X. (2019). Marriage matching and labor market performance under sex ratio imbalance: an empirical analysis based on quasi-natural experiment of one-child policy (in Chinese). World Econ. Pap. 4, 67–84.

[ref12] Li B. Qi Z. Ding R. (2018). China's potential GDP growth rate in the reform process: estimates and projections (in China). Modern Econ. Sci. 40, 1–13.

[ref13] Liu H. (2016). The influence of college enrollment expansion on the age of first marriage in China: based on census data (in Chinese). Popul. Econ. 1, 19–28. doi: 10.3969/j.issn.1000-4149.2016.01.003

[ref14] Liu B. Liu Y. (2018). Marriage effects of higher education: delaying marriage or choosing not to marry? New evidence from synthetic control methods (in Chinese). J. Shanghai Univ. Fin. Econ. 20, 93–109. doi: 10.16538/j.cnki.jsufe.2018.03.007

[ref15] Marphatia A. A. Saville N. M. Amable G. S. Manandhar D. S. Cortina-Borja M. Wells J. C.. (2020). How much education is needed to delay women's age at marriage and first pregnancy? Front. Public Health 7:396. doi: 10.3389/fpubh.2019.00396, PMID: 31993411 PMC6964653

[ref60] Michael J. B. Lee A. L. (2006). Urban-rural education gap and urban-rural income gap (in Chinese). J. Yunnan Admin. Coll. 6, 153–156. doi: 10.16273/j.cnki.53-1134/d.2006.06.042

[ref16] Parsons T. (1994). Education, Marriage, and First Conception in Malaysia. Journal of Human Resources, 29, 1167–1204. doi: 10.2307/146137

[ref17] Pei Z. (2006). Urban-rural education gap and urban-rural income gap (in Chinese). J. Yunnan Admin. Coll. 6, 153–156. doi: 10.16273/j.cnki.53-1134/d.2006.06.042

[ref18] Raymo J. M. (2003). Educational attainment and the transition to first marriage among Japanese women. Demography 40, 83–103. doi: 10.1353/dem.2003.0008, PMID: 12647515

[ref19] Requena M. Salazar L. (2014). Education, marriage, and fertility: the Spanish case. J. Fam. Hist. 39, 283–302. doi: 10.1177/0363199014527592

[ref20] Sewell W. H. Haller A. O. Ohlendorf G. W. (1970). The educational and early occupational status attainment process: replication and revision. Am. Sociol. Rev. 35:1014. doi: 10.2307/2093379

[ref21] Sewell W. H. Shah V. P. (1967). Socioeconomic status, intelligence, and the attainment of higher education. Sociol. Educ. 40, 1–23. doi: 10.2307/2112184

[ref22] Shen Y. Wu F. Zhang J. Chen L. (2013). The impact of urban and rural differences on educational development: an empirical study based on oacaxa-blinder decomposition technique. J. Agrotech. Econ. 7, 11–18. doi: 10.13246/j.cnki.jae.2013.07.011

[ref23] Wang F. Shi Y. (2014). An empirical study of family background, educational expectation and college education acquisition based on Shanghai survey data (in Chinese). Chin. J. Sociol. 1, 175–195. doi: 10.15992/j.cnki.31-1123/c.2014.01.011

[ref24] Wang P. Wu Y. (2013). Factors influencing the age of first marriage: a study based on CGSS2006. Chin. J. Sociol. 33, 89–110. doi: 10.15992/j.cnki.31-1123/c.2013.03.009

[ref25] Wen J. (2007). A dynamic study on the widening of urban and rural education inequality and income gap in China (in Chinese). Modern Econ. Sci. 29, 40–45. doi: 10.3969/j.issn.1002-2848.2007.05.006

[ref26] Wu Y. (2010). Looking for archimedes’ "lever" - is "birth quarter" a weak instrumental variable? (in Chinese). China Econ. Q. 2, 661–686. doi: 10.13821/j.cnki.ceq.2010.02.006

[ref27] Wu Y. (2012). A study on gender differences in educational attainment between urban and rural residents in China (in Chinese). Chin. J. Sociol. 32, 112–137. doi: 10.15992/j.cnki.31-1123/c.2012.04.010

[ref28] Wu Y. Liu Q. (2015). The impact of college enrollment expansion on marriage market: leftover women? Men? (in Chinese). China Econ. Q. 14, 5–30. doi: 10.13821/j.cnki.ceq.2015.01.002

[ref29] Xing C. Li S. (2011). The "great leap forward" of enrollment expansion, educational opportunities, and employment of college graduates (in Chinese). China Econ. Q. 10, 1187–1208. doi: 10.13821/j.cnki.ceq.2011.04.004

[ref30] Yang J. Huang X. (2010). The internal mechanism of education inequality and income distribution gap: an analysis based on Chinese provincial panel data (in Chinese). J. Public Manag. 3, 75–126.

[ref31] Yang Z. Zhang C. (2018). Effects of deepening education on age of first marriage and number of births: a quasi-experimental study based on compulsory education law (in Chinese). Pop. Dev. 24, 18–32.

[ref32] Zhang W. Tan X. (2021). Temporary postponement of fertility: a cognitive study of female egg freezing among marriageable young adults (in Chinese). Contemp. Youth Res. 4, 39–45. doi: 10.3969/j.issn.1006-1789.2021.04.006

[ref33] Zhu Z. Zhao C. (2019). How much does going to college delay the age of first marriage?-- estimation based on IV-Tobit model (in Chinese). Pop. J. 41, 5–16. doi: 10.16405/j.cnki.1004-129X.2019.02.001

